# First report of *Leishmania infantum* infection in the endangered orangutan (*Pongo pygmaeus pygmaeus*) in Madrid, Spain

**DOI:** 10.1186/s13071-018-2772-1

**Published:** 2018-03-20

**Authors:** Guadalupe Miró, Amelia Troyano, Ana Montoya, Fernando Fariñas, Ma Luisa Fermín, Luís Flores, Carlos Rojo, Rocío Checa, Rosa Gálvez, Valentina Marino, Cristina Fragío, Eva Martínez-Nevado

**Affiliations:** 1 Department of Animal Health, Veterinary Faculty, Universidad Complutense de Madrid, Madrid, Spain; 2 Centro de Rescate de Primates “RAINFER”, Fuente del Saz, Madrid, Spain; 3 Institute of Clinical Immunology and Infectious Diseases, Málaga, Spain; 4 Department of Animal Medicine and Surgery, Veterinary Faculty, Universidad Complutense de Madrid, Madrid, Spain; 5 Centre de Rehabilitation des Primates de Lwiro, Lwiro Village, South Kivu, Democratic Republic of Congo; 6 Zoo de Madrid, Casa de Campo s/n, 28011 Madrid, Spain

**Keywords:** Captive wild, Endangered species, *Leishmania infantum*, Leishmaniosis, Madrid, Orangutans, *Pongo pygmaeus pygmaeus*, Sand flies

## Abstract

**Background:**

Some wild animals have been recognized as potential reservoirs of *Leishmania infantum* infection (e.g. carnivores, lagomorphs, rodents, etc.). *Leishmania infantum* was also identified infecting humans and lagomorphs (i.e. hares and rabbits) over the period of 2009–2016, with the latter acting as the main reservoirs involved in the human leishmaniosis outbreak in Madrid.

**Results:**

Two cases of clinical leishmaniosis are reported in orangutans (*Pongo pygmaeus pygmaeus*) housed at two different centres in Madrid. The first is the case of a 36-year-old male orangutan with severe weight loss and apathy. A complete blood count and biochemical profile revealed anaemia, neutropenia, hypoalbuminaemia and elevated transaminases. Hepato-splenomegaly was also observed. Four months later, due to worsening of clinical signs (mainly bilateral epistaxis), blood and bone marrow samples were collected. Amastigotes of *L. infantum* were detected in macrophages from a bone marrow aspirate and by specific polymerase chain reaction. The second case was a 34-year-old female orangutan with severe weight loss and apathy and no other apparent clinical signs. A complete blood count and biochemical profile revealed anaemia, pancytopenia and hypoalbuminaemia. Splenomegaly and pericardial effusion were also observed. As leishmaniosis was included in the differential diagnosis, both blood and bone marrow samples were collected. *Leishmania infantum* infection was confirmed by microscopy, molecular diagnosis and serology (immunofluorescence antibody test). Both animals were treated daily with oral miltefosine for 28 days; allopurinol was also given uninterruptedly in Case 2 for at least 6 months. During follow-up, though good clinical recovery was clear, a lack of parasitological cure was confirmed molecularly in both blood and bone marrow samples from the two orangutans. In both habitats, the presence of the sand fly vector identified as *Phlebotomus perniciosus* was confirmed.

**Conclusions:**

To our knowledge, this is the first report of *L. infantum* infection in great apes and in the endangered species *P. p. pygmaeus*. We are presently looking for *L. infantum* in other non-human primates living in the same peri-urban areas. If detected, we will examine the impacts of this serious disease on these critically endangered species.

## Background

Zoonotic leishmaniosis due to *Leishmania infantum* (syn. *L. chagasi*) is a vector-borne disease endemic in southern Europe, Asia, North Africa and South America. In Europe, leishmaniosis is spread *via* the bites of sand flies of the genus *Phlebotomus* and the disease mainly affects domestic dogs and humans beings, the former being the main reservoir for this infection. In addition to dogs and humans, *L. infantum* infection has been reported in other European domestic and wild animals such as carnivores [cat (*Felis catus*), gray wolf (*Canis lupus*), red fox (*Vulpes vulpes*), golden jackal (*Canis aureus*), Iberian lynx (*Lynx pardinus*), genet (*Genetta genetta*), mustelids (family Mustelidae), mongoose (family Herpestidae)], lagomorphs, equines (family Equidae), wallaby (*Macropus rufogriseus rufogriseus*) and rodents) [1–7]. Some of these species are of high conservation value such as housed wallabies in which this infection could have serious impacts on morbidity and/or mortality [3], or the threatened Iberian lynx [8].

Since the first cases of *L. infantum* infection were detected in wallabies (*M. r. rufogriseus*) in Madrid [3, 9], some clinical veterinarians in zoos and wildlife parks have included leishmaniosis in their differential diagnosis protocols for animals living in endemic areas of leishmaniosis or animals that came from endemic areas. Leishmaniosis has been also in the spotlight since, in 2009, the largest human leishmaniosis outbreak in Europe affected the south-west Madrid region [10], where hares (*Lepus granatensis*) and rabbits (*Oryctolagus cuniculus*) were confirmed as reservoirs responsible for the transmission of leishmaniosis [6, 7]. However, the epidemiological role of some wildlife species remains to be established [2, 11–13].

In this study, two cases of *L. infantum* infection in non-human primates (orangutans) are described. As far as we are aware, this is the first description of *L. infantum* infection in this endangered species.

## Methods

### Study area

The two cases reported here from two orangutans (*Pongo pygmaeus pygmaeus*) housed in two different centres in Madrid, Spain. Rainfer (Primates Rescue and Rehabilitation Centre) is in the north of the Madrid Autonomous Community (40°32'38.5"N, 3°38'31.0"W). Since its founding in 1995, this centre has been involved in the rescue, rehabilitation and lifelong care of primates in Spain. The centre consists of some 4 ha in the midst of a protected natural environment and hosts 135 primates of 25 different species. The second orangutan is housed at Madrid Zoo within a forested area to the south-west of central Madrid (40°25'22.2"N, 3°45'32.0"W). At this zoological park, several protected species are bred and the park hosts over 4000 animals of more than 350 species.

### Animals

The two cases reported were both *P. p. pygmaeus*. The first case was a 36-year-old male orangutan born in 1981 in Rhenen (The Netherlands). In 1994, the animal was transferred to Spain, first to Málaga for 3 years and then in Valencia until it was moved to Rainfer (Madrid) in 2008. The second case was a female orangutan some 34-years-old. The animal was born in the Artis Amsterdam Royal Zoo (The Netherlands) and transferred to Madrid Zoo in 2009.

### Sample collection

Prior to clinical examination and sample collection, animals were anesthetised with a combination of 230 mg ketamine plus 2.3 mg medetomidine intramuscular, and oxygen and isoflurane were used as maintenance anaesthesia. Peripheral blood (5 ml) was collected from the cephalic vein into four tubes containing (i) lithium heparin (1 ml) for biochemical profile; (ii) EDTA (0.5 ml) for full blood counts and blood smears to detect the presence of any other blood parasites (e.g. *Hepatozoan* spp., *Babesia* spp., microfilariae, etc.); (iii) EDTA (1 ml) to assess *Leishmania* infection by polymerase chain reaction (PCR); and (iv) a tube without additives (2 ml) for serological tests. Blood and serum samples were kept at 4 °C until processed at the laboratory. Bone marrow from the costochondral joint or iliac crest and/or lymph node aspirates were obtained to prepare smears and were then stored in 200 μl of buffer NET 10 (NaCl 10 mM, EDTA 10 mM,Tris 10 mM). All samples for DNA extraction were stored at -20 °C until further analysis.

### Assessment of *Leishmania* infection

#### Microscopy

Giemsa-stained bone marrow smears were examined by light microscopy (×400 and ×1000 magnification) to assess the presence of *Leishmania* spp. amastigotes in macrophages.

#### Serum antibody testing

For serological tests, specific antibodies to *Leishmania* spp. were detected using the indirect immunofluorescence antibody test (IFAT) against in-house cultured promastigotes. The IFAT for anti-*Leishmania*-specific immunoglobulin G (IgG) antibodies was performed with an anti-human IgG fluorescein conjugate as described previously [14] using a cut-off of ≥ 1:100 to define seropositivity.

#### Molecular analysis

Genomic DNA was isolated from peripheral blood and tissue samples using the QIAamp® DNA mini kit (Qiagen, Hilden, Germany) according to the manufacturer’s instructions. The starting material was either 200 μl of blood or 20 mg of tissue. Obtained DNA was eluted in 200 μl molecular-grade water for blood and tissue samples. DNA samples were stored at -20 °C until use.

For *Leishmania* detection and species identification, a 20 μl aliquot of eluted DNA was used for each PCR. The parasite was detected using a nested PCR protocol by amplification of a portion of the ITS-1 gene according to the protocol described by Schönian et al. [15] but slightly modified. This protocol is *Leishmania* genus-specific and uses the primer pair LITSR (5'-CTG GAT CAT TTT CCG ATG-3')/ L5.8S (5'-TGA TAC CAC TTA TCG CAC TT-3') in the first reaction. In the second mixture, the starting primers were replaced with the primers SAC (5'-CAT TTT CCG ATG ATT ACA CC-3') and VAN2 (5'-GCG ACA CGT TAT GTG AGC CG-3'). The PCR amplification product (280–330 bp) was visualised using a dark reader trans-illuminator (Clare Chemicals, Colorado, USA).

#### DNA sequencing

*Leishmania*-positive ITS1 PCR products corresponding to the expected length were excised from agarose gels and purified using the QIAquick Gel ExtractionKit (Qiagen) as described by the manufacturer. The products were sequenced with the corresponding forward and reverse primers at the sequencing service of the Genomics department, UCM, using an ABI Prism 3730 system (Applied Biosystems, California, USA).

Sequence chromatogram files were analysed using Chromas 2.1.1 and imported into BioEdit v.7.0.5 for editing, assembly and alignments. The sequences obtained were aligned with sequences available from GenBank using Clustal W and compared with additional *Leishmania* spp. sequences available from GenBank using the BLAST program (http://blast.ncbi.nlm.nih.gov/Blast.cgi) to determine percentage identities of the generated sequences against published sequences.

### Entomological data

A preliminary entomological survey was carried out in the orangutans’ environment at the two centres. In July 2017 and in October 2017, five miniature light traps [Model 2836BQ, improved version of the mosquito light trap developed by the Centers for Disease Control (CDC)] were set-up at each site overnight. Traps were placed in the afternoon and recovered early in the morning. Collected sand flies were kept in 70% ethanol until processing. At the laboratory, females were cleared in Mark André medium [16], mounted on glass slides in Hoyer medium [17], and species identified using identification keys [18].

## Results

### Case 1

The male orangutan was examined in December 2016 due to a clinical picture characterised by severe weight loss and apathy. A complete blood count and biochemical profile revealed regenerative anaemia (red blood cell count = 2.84 × 10^12^/l; haematocrit of 25.1%), leucocytopenia (white blood cell count = 3.2 × 10^9^/l) hypoalbuminaemia (2.4 g/dl) and elevated transaminases (ALT = 51 U/l AST = 66 U/l). Hepato-splenomegaly was also observed by abdominal ultrasonography. The initial differential diagnosis included autoimmune haemolytic anaemia and vector borne diseases, and the recommended treatment was palliative consisting of oral vitamin C (500 mg/24 h), doxycycline (200 mg/24 h), folic acid (5 mg/24h), paracetamol (500 mg/12 h), omeprazole (20 mg/kg) and prednisone (35 mg/12 h). In March 2017, due to worsening of clinical signs (mainly bilateral epistaxis), blood and bone marrow samples were collected. The same haematological and biochemical abnormalities were observed as initially, and this timeleishmaniosis was included in our differential diagnosis and consequently *L. infantum* amastigotes were detected in bone marrow aspirate macrophages. Four months later (June 2017) specific PCR conducted in stored blood samples confirmed the diagnosis.

The animal was treated daily with oral miltefosine (2.5 mg/kg/day for 28 days), omeprazole, prednisone and a vitamin complex (B, C, D and K). In addition, metronidazole (250 mg) and spiramycin (1.5 M IU) combination (PO BID for 2 weeks) treatment was given for an infected tooth.

Following the miltefosine-based treatment, the animal started to gain weight and its general health condition improved. Three months later (July 2017), haematological and biochemical tests were repeated, observing normalised variables except for hepatic enzymes. Hepatomegaly was also observed by ultrasonography. The parasitological results are shown in Table 1. Currently, the animal's general clinical status is stable.

**Table 1:** Clinical signs, serology and PCR test results recorded in the two orangutans (*Pongo pygmaeus pygmaeus*) examined in this study

Animal description	Case 1, male, 36 years			Case 2, female, 34 years	
Dates of screening	December 2016 (before diagnosis)	March 2017 (diagnosis)	July 2017 (after treatment)	June 2017 (diagnosis)	October 2017 (after treatment)
Clinical signs	Weight loss, apathy, hepato-splenomegaly	Weight loss, apathy, hepato-splenomegaly, bilateral epistaxis	Hepatomegaly	Weight loss, apathy, splenomegaly, pericardial effusion	No signs, no weight gain
CBC [reference values (ZIMS)]					
Red blood count (3.62–5.89) ×10^12^/l	2.84	2.78	4.68	2.18	3.09
Haematocrit (29.0–44.9) %	25.1	21.1	41.8	16.3	20.3
Haemoglobin (8.9–13.9) g/dl	7.5	6.2	11.5	4.1	5.4
White blood count (3.8–17) ×10^9^/l	3.2	2.0	8.1	0.538	0.42
Platelets (84–309) × 10^9^/l	101	60	142	77.5	76.3
BP [reference values (ZIMS)]					
Albumin (3.0–5.2) g/dl	2.4	2.1	2.7	2.1	2.4
ALT (6–43) U/L	51	76	41	17	20
AST (4–33) U/L	66	106	33	18	37
Serology results (IFAT)	1/200	1/400	1/200	1/400	1/200
Bone marrow cytology (microscopy)	nt	POS	NEG	POS	NEG
PCR results					
Peripheral blood	POS	POS	nt	POS	POS
Bone marrow	nt	nt	POS	POS	POS

*Abbreviations*: *CBC* complete blood counts, *BP* biochemical profile, *POS* positive, *NEG* negative, *nt* not tested, *IFAT* immunofluorescence antibody test, *ZIMS* Species 360 (Zoological Information Management System 2017) [43]

### Case 2

In May 2017, the female orangutan was examined because of severe weight loss and apathy with no other apparent clinical signs. A complete blood count and biochemical profile revealed anaemia (red blood cell count = 2.18 × 10^12^/l; haematocrit of 16.3%; haemoglobin 4.1 g/dl), pancytopenia (white blood cell count = 0.538 × 10^9^/l; platelets = 77.5 × 10^9^/l) and hypoalbuminaemia (2.4 g/dl). Supportive treatment was given consisting of blood transfusion (from a healthy adult male of the same group) and omeprazole 40 mg per os (PO), once daily (SID), ferrous sulphate 80 mg PO SID, doxycycline 100 mg PO SID and 500 mg paracetamol PO BID. Splenomegaly and pericardial effusion were also observed. A second intervention was planned for bone marrow collection. Leishmaniosis was included in the differential diagnosis and both blood and bone marrow aspirates were collected. *L. infantum* infection was confirmed by microscopy, molecular diagnosis and serology (IFAT) (see Table 1).

The orangutan was treated daily with oral miltefosine (2.5 mg/kg PO SID) for 28 days, and with allopurinol (300 mg PO BID) and vitamin complex indefinitely (at least 6 months).

During follow-up (3 months later, October 2017), though good clinical recovery was evident, haematological and biochemical variables were similar to pretreatment values (red blood cell count = 3.09 × 10^12^/l; haematocrit of 20.3%; haemoglobin 5.4 g/dl; white blood count = 0.42 × 10^9^/l; platelets = 76.3 × 10^9^/l: albumin 2.4 g/dl; globulin 4.6 g/dl; albumin/globulin 0.5). A lack of parasitological cure was confirmed molecularly in both the blood and bone marrow samples (see Table 1).

Lastly, despite no apathy there was still no weight gain and we administered a second cycle of oral miltefosine (for December 2017, 4 months after the first cycle). To date, the animal continues with the same oral daily dose of allopurinol for at least 6 months.

### Sequencing results

The concatenated ITS sequence was 259 and 260 bp for Case 1 and 2, respectively. Both sequences were 99% identical to the *L. infantum* isolate MHOM/ES/87/Lombardi strain sequence (GenBank: AJ000295). These results are consistent with the infection of both orangutans with this strain. This causative agent was recently identified in Bennett’s wallabies (*M. r. rufogriseus*) kept in a wildlife park in Madrid, Spain [3] and in reported cases of the disease in humans and hares as the result of the leishmaniosis outbreak in the Madrid region [19].

### Entomological data

In both habitats, the presence of the sand flies was confirmed. At Rainfer (habitat of Case 1), 7 sand fly specimens were captured on October 3rd around the orangutan’s habitat: 4 *Phlebotomus perniciosus* (1 female and 3 males) and 3 *Sergentomyia minuta* (2 females and 1 male). At the Madrid Zoo (habitat of Case 2), 17 sand fly specimens were captured on July 10th around the orangutan’s habitat: 15 *P. perniciosus* (3 females and 12 males), 1 *Phlebotomus papatasi* (1 male) and 1 *S. minuta* (1 male). The most frequent vector of *L. infantum* in Spain, *P. perniciosus*, was detected in the two places surveyed. We observed one engorged *P. perniciosus* female at Rainfer and another one at the Madrid Zoo.

## Discussion

To our knowledge, this report describes the first two clinical cases of leishmaniosis in orangutans (*P. p. pygmaeus*) and is the first notification of *L. infantum* infection in non-human primates (NHP) in Spain. Descriptions exist in Brazil of *Leishmania* infection in NHP. In one report, a black-fronted titi monkey (*Callicebus nigrifrons*) developed a fatal disease with clinical signs and lesions compatible with leishmaniosis; *L. infantum* (syn*. L. chagasi*) was confirmed by PCR and immunohistochemistry [12]. In another case in Bauru, São Paulo, Brazil, *Leishmania amazonensis* was detected by molecular methods in blood samples from a captive spider monkey (*Ateles paniscus*), which showed weight loss and pale mucous membranes [20].

Malta et al. [12] also detected *L. infantum* by PCR in blood samples from NHP housed in a zoo in Belo Horizonte (State of Minas Gerais, Brazil): six black-fronted titi monkeys (*C. nigrifrons*), one howler monkey (*Alouatta guariba*), three golden-bellied capuchins (*Cebus xanthosternos*), one golden-headed lion tamarin (*Leontopithecus crysomelas*), one black-headed owl monkey (*Aotus nigriceps*), two Rio Tapajos’ sakis (*Pithecia irrorata*) and three emperor tamarins (*Saguinus imperator*). These 17 NHP showed no clinical signs of disease. As indicated in a study conducted by Carneiro et al. [21], these infected primates were clinically healthy. Carneiro et al. hypothesized that New World primates have developed an innate immune response mechanism capable of controlling macrophage intracellular growth of *L. infantum* [21]. However, several field studies have confirmed a high susceptibility to *Leishmania* spp. infection with cutaneous signs in owl monkeys (*Atous trivirgatus*) and Geoffroy’s tamarins (*Saguinus geoffroyi*) [22], while in tufted capuchin monkeys (*Cebus paella*) and bearded sakis (*Chiropotes satanus*), visceral leishmaniosis [23, 24] was confirmed. In addition, experimental studies have observed fulminating visceral leishmaniosis both in Neotropical [owl monkeys (*Atous trivirgatus*), tufted capuchin monkeys (*Cebus paella*), (*Callithrix jacchus jacchus*), squirrel monkeys (*Saimiri sciureus*)] and Old World monkeys [common marmoset vervet monkeys (*Cercopithecus aethiops*), rhesus macaques (*Macaca mullata*), and langur monkeys (*Presbytis entellus*)] [25–31].

Despite our confirmation of two cases of clinical leishmaniosis in orangutans in Madrid, the role of this species in transmitting this zoonotic disease will remain unknown until future xenodiagnostic studies are carried out.

Our results indicate the high presence of *Leishmania* in the environment (there may be many potential reservoirs and sand flies sharing the same habitat), since two orangutans became infected. Accordingly, there will be a risk of *L. infantum* transmission to other susceptible animals living in nearby areas as well as to humans. After all, these animals are living in an urban environment in close contact with humans, possibly posing a public health risk. Some authors have argued that wild animals may act as sentinels indicating the risk of zoonoses, and highlighting the importance of the ‘One Health’ concept [32–35].

Our preliminary entomological survey revealed the presence of *P. perniciosus* in the nearby environment of the orangutans. A more exhaustive survey is needed during the active period of this phlebotomine. This would enable us to look for infected phlebotomines in larger numbers of captured *P. perniciosus* at both sites and determine their blood-feeding preferences on several possible hosts by using molecular tools.

Orangutans are considered critically endangered species according to the IUCN [36]. Both of the present orangutans are included in the EEP (European Endangered Species Programme) of EAZA (European Association of Zoos and Aquaria) [36] targeted at maintaining healthy populations of animals in captivity while safeguarding their genetic health. The morbidity and mortality of these animals is not only a dramatic situation for an animal reserve or zoo, but also for the future breeding and conservation of these species [37]. Efforts are thus needed to avoid infection by *L. infantum* in these species through early diagnostics and the use of preventative measures in these environments as well as on animals (e.g. topical pyrethroid formulations). More extensive entomological surveys are needed to effectively design insecticide control measures applied to the environment. Besides, in captive wild animals, it is also necessary to control stress and any disease that could compromise the immune system and lead to clinical leishmaniosis in infected animals [12].

Although, there are no data available regarding the treatment of leishmaniosis in NHP, we opted for miltefosine due to its easier administration (oral route) and its effectiveness observed in human medicine studies for the treatment of visceral leishmaniosis [38]. We should not, however, forget about the risk of resistance when this drug is used as monotherapy, as suggested in studies conducted in India [39, 40] and in immunocompromised patients [41, 42].

Finally, we should highlight the importance of including *L. infantum* infection in the differential diagnosis list for captive wild animals (especially endangered species) with clinical signs or laboratory abnormalities compatible with this disease living in endemic areas.

## Conclusions

To our knowledge, this is the first report worldwide of *L. infantum* infection in great apes and in *Pongo pygmaeus pygmaeus.* As the presence of the sand fly vector was also confirmed in the orangutans’ habitat, our results suggest the possible detection of *L. infantum* in other non-human primates living in this endemic area, which may be a risk for endangered species living in captivity.

## Abbreviations

ALT: Alanine aminotransferase
AST: Aspartate aminotransferase
BID: Twice a day
EAZA: European Association of Zoos and Aquaria
EDTA: Ethylenediaminetetra-acetic acid
EEP: European Endangered Species Programme
g/dl: grams per decilitre
IFAT: Indirect immunofluorescence antibody test
IU: International units
NHP: Non-human primate
PCR: Polymerase chain reaction
PO: *Per os*
SID: Once a day
U/l: Unit per litre
